# Supplementary figures and images for: Preparation and Application of Quaternized Chitosan- and AgNPs-Base Synergistic Antibacterial Hydrogel for Burn Wound Healing
Source: Molecules. 2021 Jul 1;26(13):4037. doi: 10.3390/molecules26134037 (PMC8271850; doi:10.3390/molecules26134037)

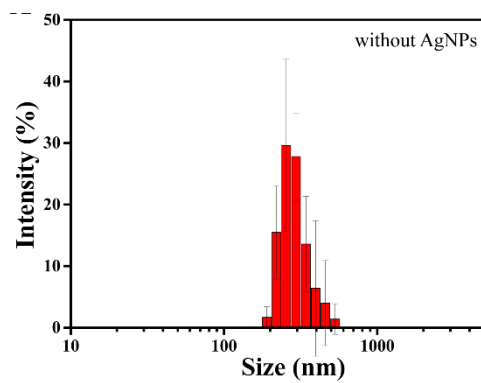

**Figure S1.** DLS of hydrogel solution without AgNPs.

Supplement: Supplementary file 1 [file molecules-26-04037-s001.zip › molecules-1246407-supplementary.pdf]
